# Dual-task tests discriminate between dementia, mild cognitive impairment, subjective cognitive impairment, and healthy controls – a cross-sectional cohort study

**DOI:** 10.1186/s12877-020-01645-1

**Published:** 2020-07-29

**Authors:** Hanna B. Åhman, Ylva Cedervall, Lena Kilander, Vilmantas Giedraitis, Lars Berglund, Kevin J. McKee, Erik Rosendahl, Martin Ingelsson, Anna Cristina Åberg

**Affiliations:** 1 Department of Public Health and Caring Sciences, Geriatrics, Uppsala University, Box 564, SE-751 22 Uppsala, Sweden; 2 School of Education, Health and Social Studies, Dalarna University, Falun, Sweden; 3 Department of Community Medicine and Rehabilitation, Physiotherapy, Umeå University, Umeå, Sweden

**Keywords:** Dual-task, Dementia, Mild cognitive impairment, Subjective cognitive impairment, Gait

## Abstract

**Background:**

Discrimination between early-stage dementia and other cognitive impairment diagnoses is central to enable appropriate interventions. Previous studies indicate that dual-task testing may be useful in such differentiation. The objective of this study was to investigate whether dual-task test outcomes discriminate between groups of individuals with dementia disorder, mild cognitive impairment, subjective cognitive impairment, and healthy controls.

**Methods:**

A total of 464 individuals (mean age 71 years, 47% women) were included in the study, of which 298 were patients undergoing memory assessment and 166 were cognitively healthy controls. Patients were grouped according to the diagnosis received: dementia disorder, mild cognitive impairment, or subjective cognitive impairment. Data collection included participants’ demographic characteristics. The patients’ cognitive test results and diagnoses were collected from their medical records. Healthy controls underwent the same cognitive tests as the patients. The mobility test Timed Up-and-Go (TUG single-task) and two dual-task tests including TUG (TUGdt) were carried out: TUGdt naming animals and TUGdt months backwards. The outcomes registered were: time scores for TUG single-task and both TUGdt tests, TUGdt costs (relative time difference between TUG single-task and TUGdt), number of different animals named, number of months recited in correct order, number of animals per 10 s, and number of months per 10 s. Logistic regression models examined associations between TUG outcomes pairwise between groups.

**Results:**

The TUGdt outcomes “animals/10 s” and “months/10 s” discriminated significantly (*p* < 0.001) between individuals with an early-stage dementia diagnosis, mild cognitive impairment, subjective cognitive impairment, and healthy controls. The TUGdt outcome “animals/10 s” showed an odds ratio of 3.3 (95% confidence interval 2.0–5.4) for the groups dementia disorders vs. mild cognitive impairment. TUGdt cost outcomes, however, did not discriminate between any of the groups.

**Conclusions:**

The novel TUGdt outcomes “words per time unit”, i.e. “animals/10 s” and “months/10 s”, demonstrate high levels of discrimination between all investigated groups. Thus, the TUGdt tests in the current study could be useful as complementary tools in diagnostic assessments. Future studies will be focused on the predictive value of TUGdt outcomes concerning dementia risk for individuals with mild cognitive impairment or subjective cognitive impairment.

## Introduction

Dementia disorders are the leading cause of disability and dependency among older adults [1], and as the proportion of older adults in the population increases, so does the global prevalence of dementia disorders [2]. Dementia disorders involve a range of cognitive and behavioral symptoms that interfere with the ability to perform daily life activities [3] and may be preceded by less severe cognitive impairment diagnoses. Mild cognitive impairment (MCI), which signifies a decline in cognitive function beyond typical aging but without having an impact on functional activities [4], and subjective cognitive impairment (SCI), which involves only a subjective reduction of cognitive function [5], are possible early manifestations of dementia disorders [5, 6]. The annual conversion rate of MCI to dementia is approximately 10 to 15% in clinical samples [7], while the corresponding number for individuals with SCI is approximately 2% [8].

Identification of dementia disorders at an early stage is needed to enable pharmacological treatment and lifestyle consultation upon diagnosis, as well as allowing the patient to make arrangements for future needs [9]. Regarding Alzheimer’s disease (AD), which explains 60–70% of all dementia disorder cases [1], early and accurate diagnosis will be of even greater importance once disease-modifying drugs are developed that can be introduced before pathologic changes become extensive [10]. However, since normal aging generally involves a decline in cognitive abilities such as mental speed [11], executive function [12], and episodic memory [13], discriminating cognitive impairment from normal aging can be challenging. That is one reason why dementia disorders are under-diagnosed [14]. The diagnostic assessment in specialist clinics may be extensive, involving neuropsychological, invasive, and imaging methods [15]. Even when using all methods available, distinguishing between mild AD and MCI, as well as between MCI, SCI and normal aging, can be difficult [4, 16, 17]. New, non-invasive and less time-consuming methods that can enhance discrimination between these diagnoses have been called for [9]. Such methods could be useful either as frontline screening tools or as diagnostic tools aimed at facilitating early and potentially more effective interventions. Different kinds of dual-task tests have been suggested for these purposes [9, 18, 19].

Dual-task testing challenges attentional capacities by the simultaneous performance of two tasks. Dual-task tests that include both gait and verbal tasks may entail outcomes that vary depending on cognitive capacity [20, 21]. Such tests commonly involve straight-line walking [18, 22] or the mobility test Timed Up-and-Go (TUG) [19, 23], combined with an attention-demanding verbal task [24]. Research has primarily focused on investigating outcomes derived from gait performance, while verbal outcomes are less explored [25].

Previous studies investigating dual-task tests for discrimination between dementia disorder and different diagnoses of cognitive impairment have shown promising results for various outcomes: test time score [19, 23], gait velocity [22, 26–29], stride time [27, 28], stride variability [27, 28], and the relative time difference between single- and dual-task performance i.e. dual-task cost [18, 19, 29]. Individuals with SCI are rarely included in such dual-task research [18, 30], despite being at a twofold risk of developing a dementia disorder compared to individuals without subjective complaints [8]. To our knowledge, there has been no previous dual-task study that has included participants across the full spectrum of diagnoses from dementia disorder through MCI to SCI, as well as cognitively healthy controls.

The aim of this cross-sectional study was to determine whether various TUG dual-task (TUGdt) outcomes discriminate between individuals with a dementia disorder, MCI, SCI, and healthy controls.

## Methods

### Setting and participants

**Fig. 1:**
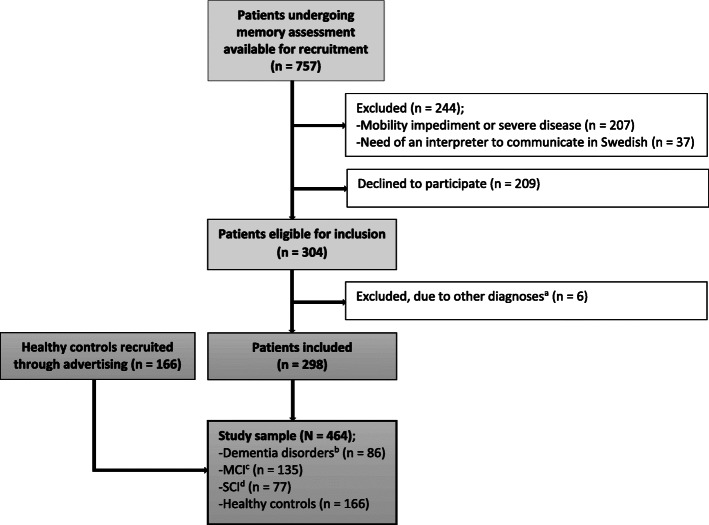
Flow chart of inclusion and exclusion. ^a^Other diagnoses: Malignant neoplasm of frontal lobe (*n* = 1); Unspecified personality and behavioural disorder due to known physiological condition (*n* = 1); Disorientation, unspecified (*n* = 1); Major depressive disorder, single episode, unspecified (*n* = 1); Idiopathic normal pressure hydrocephalus (*n* = 1); Multiple sclerosis (*n* = 1). ^b^Alzheimer’s disease (*n* = 50); Other dementia disorders (*n* = 36). ^c^Mild cognitive impairment. ^d^Subjective cognitive impairment

The current study forms part of the Uppsala-Dalarna Dementia and Gait (UDDGait) project. UDDGait is an ongoing, longitudinal, prospective cohort study, in which patients have been consecutively included when undergoing memory assessment at two specialist clinics in Sweden during the study recruitment period (April 2015 to February 2017 at Uppsala University Hospital and June 2015 to June 2016 at Falu Hospital, with exceptions of regular vacations). Excepting those patients whose appointments were booked at short notice or who could not be included for other administrative reasons, 757 patients were available for recruitment. The exclusion criteria were: inability to walk three meters back and forth or to rise from a sitting position, indoor use of a walking aid, current or recent hospitalization (within the last 2 weeks), or need of an interpreter to communicate in Swedish. Individuals without cognitive impairment served as healthy controls and were recruited through advertisements and flyers (May 2017 to March 2019 in Uppsala). The inclusion criteria for the healthy controls were a subjective perception of normal cognitive function and a Mini Mental State Examination (MMSE) score of > 26. The exclusion criteria were the same as for the patients as described above. The total study sample consisted of 464 participants (see Fig. 1).

Ethical approval was granted from the Regional Ethical Review Board in Uppsala. Informed consent was attained from all participants during enrollment.

### Data collection

The data collection procedures used in UDDGait have been described previously [31]. The patients’ diagnostic assessments and the TUG tests were blinded since the diagnoses were not known when the TUG tests were performed. All participants reported demographic characteristics including educational level (university education or not). The patients underwent a clinical diagnostic assessment led by a geriatrician, and the healthy controls carried out the same clinical cognitive tests as the patients (Table 1). The TUG tests were then performed. Additionally, for descriptive purposes, all participants carried out short versions of the Geriatric Depression Scale [32] and the General Motor Function Assessment Scale [33, 34], as well as static balance according to the Bohannon Method [35], and handgrip strength using a dynamometer [36] (Additional file 1).

After all tests had been carried out and the diagnoses were set, the patients’ diagnostic information was collected from their medical records, so all patients were allocated to one of the three diagnostic groups: dementia disorders, MCI, or SCI. Patients with other diagnoses were excluded (*n* = 6) (Fig. 1).

### Clinical diagnostic assessment

The diagnostic procedure was part of the clinical routine for patients undergoing memory assessment and involved a geriatrician’s careful evaluation of the patient’s history, structural brain imaging, and cognitive testing [37] (MMSE, Clock Drawing Test, Verbal Fluency Test, and Trail Making Test A and B). When considered relevant, supplemental assessments such as neuropsychological testing and cerebrospinal fluid analysis were carried out. A geriatrician diagnosed the patients based on established criteria [4, 38–42].

### Timed Up-and-Go single- and dual-task tests

The mobility test TUG (TUG single-task, TUGst) evaluates functional mobility through observation and timing of a test person rising from an armchair, walking three meters at a comfortable pace, turning around, walking back, and sitting down again [43]. The TUGst time score is independently associated with multiple cognitive domains, possibly explained by the test involving both transfers, walking, and turning [44].

The dual-task test procedure used in the current study was previously tested in a pilot study, which resulted in improvements to the test procedures [45]. In the current study, five physical therapists were trained to lead the standardized test procedure, and led all TUG tests. Before TUG data collection started, the test leader gave the participant standardized instructions and showed how to perform TUGst, and the participant had one trial to familiarize him/herself with the test. The recorded data collection consisted of three different TUG tests; first TUGst, followed by TUG while simultaneously naming different animals (TUGdt NA), and finally TUG while simultaneously reciting months in reverse order (TUGdt MB). The choice of the verbal task NA was based on previous research [29, 45, 46], while to the best of our knowledge MB has not been used as a part of dual-task research [45]. Participants were instructed to execute all TUG tests at their own pace, with the simultaneous performance of the verbal task at a self-selected speed. In order to standardize the test procedure and to make the test situation less stressful for the participant, the test leader instructed participants to keep walking even if they could not think of anything to say. During the testing, the test leader answered spontaneous questions from the participants on how to execute the tests. Apart from that, complementary instructions were only given when participants kept walking without turning at the 3-m marking on the floor or when they returned to the chair but did not sit down, so that the tests could be completed. When participants performed the verbal tasks in a way that clearly showed that they had misunderstood the instructions (e.g. made animal sounds instead of naming animals), the instructions were repeated and they were asked to restart. A stopwatch with an accuracy of 0.01 s was used to time the TUG tests, from the participants standing up (back leaving the backrest), to sitting down (posterior touching the seat). Two video cameras, one placed in front and one to the side of the setup, recorded the tests to capture the verbal performance for the current study, and the mobility performance for future investigations.

### Quantification of Timed Up-and-Go dual-task test outcomes

Using the video cameras’ sound recordings, the number of different animals recited during TUGdt NA and the number of months recited in correct order during TUGdt MB were counted and registered, which another researcher validated by independently performing the same procedure. In order to capture both the mobility and the verbal performance of the dual-task tests, each participant’s average number of correct words (animals and months) recited per time unit during the TUGdt tests was calculated. The measures “animals/10 s” and “months/10 s” were calculated as 10*(TUGdt number of words/TUGdt time score). Dual-task cost, i.e. the relative time difference between TUGst and TUGdt, was calculated as 100*(TUGdt time score –TUGst time score)/TUGst time score.

### Statistical analysis

Participants’ characteristics were summarized using means and standard deviations or frequencies and percentages. The test outcomes were not normally distributed and are therefore presented as medians with interquartile ranges. Minimum and maximum values concerning the TUG test outcomes are also given (Table 1).

**Table 1:** Overview of Participant Characteristics and Test Results^i^

**Characteristic**	**Total Sample (*N*** **= 464)**	**Dementia Disorders (*n*** **= 86)**	**MCI (*n*** **= 135)**	**SCI (*n*** **= 77)**	**Healthy Controls (*n*** **= 166)**
Age, years, mean +/− SD	71 +/− 10	76 +/− 8	73 +/− 9	67 +/− 9	70 +/− 11
(Min.-max.)	(39–94)	(55–94)	(49–91)	(39–85)	(50–91)
Female, n (%)	217 (47)	37 (43)	59 (44)	36 (47)	85 (51)
Married or cohabiting, n (%)	317 (68)	60 (70)	88 (65)	51 (66)	118 (71)
University educated, n (%)	241 (52)	32 (37)	55 (41)	33 (43)	121 (73)
**Clinical Cognitive Test Result**^a^					
MMSE, score	28 (25–29)	22 (20–25)	26 (24–28)	29 (28–30)	29 (29–30)
Clock Drawing test^b^, score	7 (6–7)	4 (2–6)	7 (6–7)	7 (7–7)	7 (7–7)
Verbal Fluency test^c^, score	19 (13–24)	11 (8–14)	15 (12–19)	21 (17–25)	24 (20–29)
TMT A^d^, passed^e^, n (%)	441 (97)	73 (87)	131 (99)	72 (100)	165 (99)
TMT B^f^, passed^e^, n (%)	305 (68)	12 (15)	70 (53)	68 (92)	155 (93)
**TUG Test Results**					
TUG single-task, s	11.5 (9.9–14.0)	14.9 (12.5–16.9)	12.6 (11.0–14.8)	10.9 (9.7–12.4)	10.1 (9.0–11.4)
(Min.-max.)	(6.1–29.9)	(7.9–28.5)	(7.4–29.9)	(7.9–26.5)	(6.1–24.1)
TUGdt NA^g^, s	13.2 (10.7–16.1)	16.7 (13.7–20.2)	13.9 (11.8–16.7)	12.1 (10.6–15.0)	11.0 (9.8–13.8)
(Min.-max.)	(5.8–40.0)	(8.9–40.0)	(7.4–35.4)	(8.0–28.3)	(5.8–26.7)
TUGdt MB^h^, s	13.3 (10.9–16.7)	17.4 (14.4–22.2)	14.2 (12.3–18.1)	12.4 (10.9–15.0)	11.1 (9.6–14.0)
(Min.-max.)	(6.1–55.0)	(9.4–55.0)	(7.4–44.4)	(8.0–28.9)	(6.1–25.8)
TUGdt NA^g^ cost, %	11.3 (3.3–21.7)	13.1 (3.3–26.3)	11.3 (2.9–19.0)	11.7 (4.7–16.5)	9.9 (2.7–23.0)
(Min.-max.)	(−18.7–148.7)	(− 10.5–148.7)	(−18.7–102.2)	(− 10.2–69.0)	(−10.2–100.5)
TUGdt MB^h^ cost, %	13.1 (3.4–28.9)	16.3 (6.6–46.7)	18.3 (3.1–31.2)	7.5 (3.3–19.3)	11.5 (3.1–23.8)
(Min.-max.)	(−21.6–293.9)	(− 18.7–293.9)	(−21.6–83.2)	(−9.0–114.3)	(−17.9–113.4)
TUGdt NA^g^, number of animals	7 (5–8)	4 (3–6)	6 (5–7)	7 (6–8)	8 (7–9)
(Min.-max.)	(0–15)	(0–10)	(0–10)	(2–12)	(3–15)
TUGdt MB^h^, number of months	7 (4–9)	4 (2–6)	6 (4–8)	8 (6–9)	9 (8–11)
(Min.-max.)	(0–13)	(0–12)	(0–12)	(2–12)	(3–13)
TUGdt NA^g^, animals/10 s	5.1 (3.3–6.8)	2.5 (1.7–3.6)	4.2 (3.0–5.6)	5.3 (4.3–7.0)	6.7 (5.7–8.3)
(Min.-max.)	(0–12.4)	(0–11.1)	(0–9.1)	(1.1–11.0)	(2.1–12.4)
TUGdt MB^h^, months/10 s	5.5 (2.9–8.0)	1.8 (0.9–3.2)	4.3 (2.2–6.1)	5.6 (4.3–8.0)	7.8 (6.4–9.4)
(Min.-max.)	(0–14.0)	(0–8.8)	(0–9.3)	(1.0–14.0)	(2.9–13.3)

*MCI* mild cognitive impairment, *SCI* subjective cognitive impairment, *Min.* minimum, *Max.* maximum, *SD* standard deviation, *MMSE* Mini Mental State Examination, *TMT* Trail Making Test, *TUG* Timed Up-and Go, *TUGdt* TUG dual-task, *TUGdt NA* Timed Up-and-Go dual-task naming animals, *TUGdt MB* Timed Up-and-Go dual-task months backwards

^a^Parts of the diagnostic assessment

^b^Missing values, *n* = 4

^c^Missing values, *n* = 19

^d^Missing values, *n* = 9

^e^Test completed within 240 s and with a maximum of four errors

^f^Missing values, *n* = 14

^g^Missing value due to discontinuing, *n* = 1

^h^Missing values due to discontinuing, *n* = 6

^i^Results are presented as median (interquartile range) if not stated otherwise

Using logistic regression models, associations were examined between the TUG-related outcomes pairwise between groups. Because the dementia disorders group comprised various dementia diagnoses, analyses examined possible group differences between AD (*n* = 50) and other dementia disorders (*n* = 36) regarding TUG test outcomes. Results were expressed as standardized odds ratios (ORs) with 95% confidence intervals. For the time scores of TUGst, TUGdt NA, and TUGdt MB, as well as TUGdt NA cost and TUGdt MB cost, the ORs express the risk increase per one standard deviation *increase* of the variable, whereas for the number of animals and number of months, as well as “animals/10 s” and “months/10 s” the ORs express the risk increase per one standard deviation *decrease* of the variable. Analyses were adjusted for participant age as a continuous variable, as well as for gender and educational level. The areas under the Receiver Operating Characteristics curves (c-statistics) were used to determine the discriminatory capacity of the TUGdt test outcomes by the adjusted logistic regression models.

Statistical tests were two-tailed and the significance level was set at *p* < 0.05. In order to account for multiple group comparisons, Bonferroni correction was applied for the comparisons dementia disorders vs. MCI, MCI vs. SCI, and SCI vs. healthy controls, i.e. three comparisons. Thus, the critical *p*-value used was 0.05/3 = 0.0167. Analyses were carried out using SPSS version 25 (IBM Corp., Armonk, NY, USA), and SAS® version 9.4 (SAS Institute Inc., Cary, NC, USA).

## Results

Participants’ demographic characteristics and test results are summarized in Table 1.

The mean age of the total sample (*N* = 464) was 71 years, and 47% of participants were women. The dementia disorders group consisted of AD (*n* = 50), unspecified dementia (*n* = 19), frontotemporal dementia (*n* = 5), vascular dementia (*n* = 4), Lewy body dementia (*n* = 4), Parkinson’s disease dementia (*n* = 2) and alcohol dementia (*n* = 2). Comparisons between AD (*n* = 50) and other dementia disorders (*n* = 36) showed no group differences regarding any of the TUG test outcomes (*p* = 0.07–0.50).

**Fig. 2:**
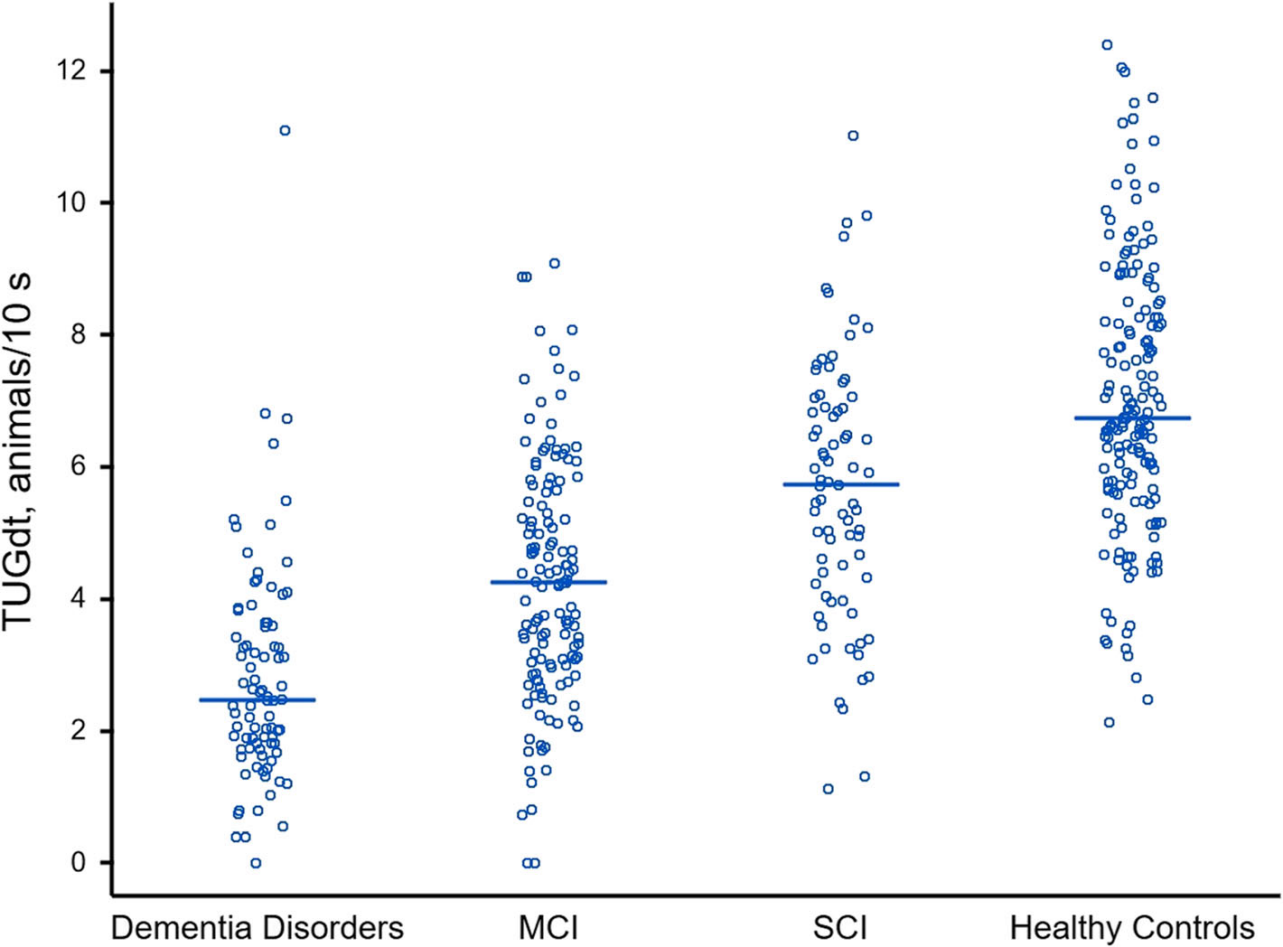
Distribution of results for TUGdt “animals/10 s” during Timed Up-and-Go dual-task naming animals. Horizontal lines on the graph show median values. TUGdt = Timed Up-and-Go dual-task; MCI = Mild cognitive impairment; SCI = Subjective cognitive impairment

All groups took longer to perform the TUGdt tests than the TUGst test. The dementia disorders group took the longest to perform the TUGst test and TUGdt tests, followed by the MCI group, the SCI group, and the healthy controls (Table 1). Following the same order across groups, the number of animals and months recited, as well as “animals/10 s” and “months/10 s” were lowest in the dementia disorders group, followed by the MCI-group, the SCI-group, and highest in the healthy controls. Similarly, individuals with dementia disorder named a median of 2.5 animals/10 s and 1.8 months/10 s, whereas individuals with MCI named 4.2 animals/10 s and 4.3 months/10 s, individuals with SCI named 5.3 animals/10 s and 5.6 months/10 s, and healthy controls named 6.7 animals/10 s and 7.8 months/10 s (Table 1). The ranges of “animals/10 s” and “months/10 s” were wide within groups, exemplified by “animals/10 s” in Fig. 2. The TUGdt NA cost and TUGdt MB cost were the only TUG test outcomes that did not follow the group order described above, not varying across groups according to the level of cognitive function (Table 1).

**Fig. 3:**
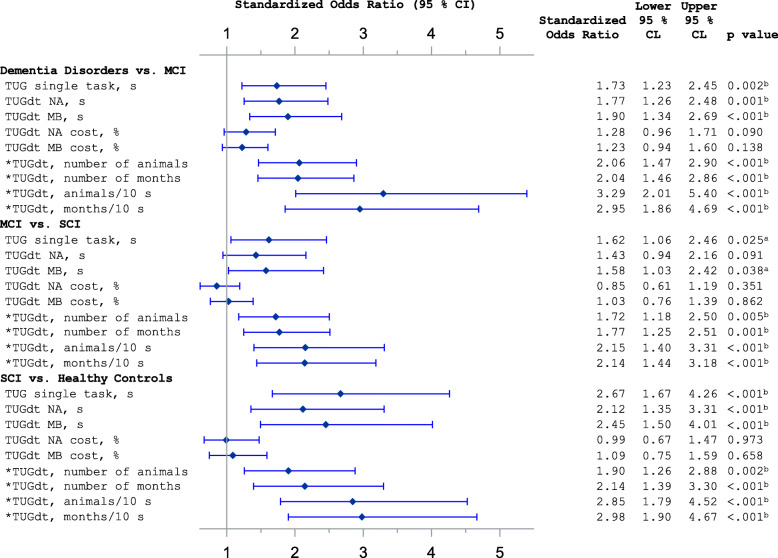
Forest plot of logistic regression models. MCI = mild cognitive impairment; SCI = subjective cognitive impairment; TUG = Timed Up-and-Go; TUGdt = Timed Up-and-Go dual-task; TUGdt NA = Timed Up-and-Go dual-task naming animals; TUGdt MB = Timed Up-and-Go dual-task months backwards. Models are adjusted for participant age, gender, and educational level. Standardized odds ratios measure risk increase per one standard deviation *increase* of the predictor or *risk increase per one standard deviation *decrease* of the predictor. ^a^Statistically significant if *p* < 0.05. ^b^Statistically significant with Bonferroni adjustment if *p* < 0.0167

The logistic regression models showed that “animals/10 s” and “months/10 s” had high ORs in discriminating between all groups. For each comparison, “animals/10 s” and “months/10 s” resulted in similar ORs. Figure 3 shows the comparisons of adjacent groups regarding cognitive function, where e.g. “animals/10 s” had an OR = 3.3 (95% CI 2.0–5.4) between dementia disorders vs. MCI, OR = 1.7 (95% CI 1.2–2.5) between MCI vs. SCI, and OR = 2.8 (95% CI 1.8–4.5) between SCI vs. healthy controls. In comparisons between the adjacent groups, the c-statistics for “animals/10 s” and “months/10 s” were between 0.72–0.79.

When comparing groups with more diverse cognitive function, “animals/10 s” had an OR = 6.0 (95% CI 3.1–11.6) between dementia disorders vs. SCI, OR = 22.3 (95% CI 9.9–50.2) between dementia disorders vs. healthy controls, and OR = 7.1 (95% CI 4.3–11.6) between MCI vs. healthy controls (Additional file 2). Furthermore, the number of animals as well as the number of months recited discriminated between all groups. The time scores of TUGst, TUGdt NA, and TUGdt MB did not discriminate between MCI vs. SCI, but between all other groups. The dual-task cost of TUGdt NA or TUGdt MB did not discriminate between any groups (Fig. 3).

## Discussion

Our results show that the TUGdt test outcomes “animals/10 s” and “months/10 s” demonstrated a high level of discrimination between dementia disorders, MCI, SCI, and healthy controls. “Words per time unit”, as applied in the current study, takes both mobility and verbal performance into consideration, which adds value to the dual-task tests. Our presentation of results focuses on discriminating between adjacent groups regarding cognitive function since these are naturally the most challenging to discriminate between in clinical assessment. Nevertheless, strong associations were shown even in the comparisons of dementia disorders vs. MCI, MCI vs. SCI, and SCI vs. healthy controls, by “animals/10 s” and “months/10 s”.

The novel TUGdt test outcome “words per time unit” summarizes the performance of both tasks included, of which the attentional load may affect one or both. Presumably, the outcome “words per time unit” eliminates the effect of one task being prioritized over the other. Dual-task research has previously focused on investigating gait-related outcomes in discriminating groups with different degrees of cognitive impairment, most likely because the means for measuring verbal outcomes have been lacking [47, 48]. From a clinical point of view, it should be important to evaluate the performance of both included tasks when using dual-task tests, since intentional or unintentional strategies may influence either the mobility or verbal performance. To our knowledge, the outcome “words per time unit” has rarely been used for this purpose, although it has been used with other test tasks [25, 49]. In one study, straight-line walking and the verbal tasks “counting backward from 50 by 2s” and “naming animals” were used, and both “numbers/s” and “animals/s” were lower for individuals with cognitive impairment compared with those without cognitive impairment, when groups were stratified by means of a MMSE cut-off score of 25 [25]. In another study, TUG and the verbal task “countdown from 50” were used, where “numbers/s” differed between individuals with AD and controls, but did not differ between AD and MCI, or between MCI and controls [49].

It is to be noted that dual-task cost, i.e. the relative time difference between single- and dual-task performances, did not discriminate between any of the groups in the current study. In previous studies, dual-task cost has been found to discriminate between mild AD and healthy controls [46] and between MCI and healthy controls [19, 29], and has shown inconsistent results when discriminating between dementia disorders and MCI [18, 19], and was not able to discriminate between MCI and SCI [18]. It may be argued that dual-task cost cannot differentiate between transitional diagnoses, because it captures subtle pathological changes and is therefore better considered an indicator of imminent cognitive decline [50]. In studies that show strong discriminative capacity of dual-task cost, straight-line walking has been used [18, 29, 46]. In studies such as ours, where dual-task tests involve TUG, however, the TUGst test alone challenges executive functions [44, 51], and this may be why the relative time difference between single- and dual-task tests is too small to be a reliable measure. Furthermore, in the current study, participants were instructed to keep walking if they did not know what to say, which most likely lowered the dual-task time scores, and thereby reduced the time difference. This instruction was given in order to standardize the test procedure and make the test less stressful for the participants as they could complete the test without feeling they had failed. In addition, the instruction was given to make the test more clinically feasible. Participants would presumably prioritize either walking speed or verbal performance without this instruction [25], and for those prioritizing the verbal performance, time scores could be extended. In previous dual-task studies, there are commonly no instructions given concerning prioritizing [19, 23, 25–29]. Alternatively, the instruction to prioritize both tasks equally has been used in order to replicate everyday life [18].

Our results showed that the TUGst time score discriminated between all groups with the exception of MCI vs. SCI. The capability of TUGst to discriminate between groups of different cognitive function is not surprising since TUGst time score is associated with global cognitive function, attention, processing speed, and memory [44]. Nevertheless, the TUGdt test outcomes “animals/10 s” and “months/10 s” appeared to show stronger associations than the TUGst time score for each comparison. This is in agreement with the underlying theory of dual-task testing which implies that two simultaneously performed tasks interfere and compete with each other for brain cortical resources [20]; a process that may be influenced by aging, neurodegenerative, and microvascular mechanisms [50]. Consequently, in dual-task testing, the attentional load of both included tasks matter and may affect the outcomes [24]. For that reason, the load of the verbal task is recommended to be chosen at or near the participants’ threshold of ability [27] without causing undue stress [20]. In our study, the verbal tasks were based on two established tests of cognitive function: The Verbal Fluency test of naming animals [52] and the Months Backward test [53], both thought to be suitably challenging for the current participants. Moreover, these verbal tasks were chosen to reflect different central functions [52, 53]. However, even though the two TUGdt tests were based on different verbal tasks, the associations with the outcomes in terms of “words per time unit” were at similar levels. Thus, both TUGdt tests appear to be equally useful in discriminating between the investigated groups.

The current study has some limitations that need to be considered. It was not possible to include all patients undergoing memory assessment in the two specialist clinics during the recruitment period. Thus, the study sample may have been biased. Additionally, the dementia disorders group in the current sample comprised different dementia diagnoses, however, analyses showed no group differences between AD and other dementia disorders regarding TUG test outcomes. The consecutive inclusion of patients undergoing memory assessment with the addition of healthy controls are strengths of our study that enabled comparisons across a wide spectrum of cognitive function. The blinded testing procedure, ensuring that the TUG tests were carried out independently of the diagnostic assessments, has minimized the risk of observer bias. Furthermore, the TUG test procedure was standardized and verbal performances were carefully validated based on video recordings. Finally, by adding Bonferroni correction to our analyses, the risk of an inflated type I error due to multiple testing was reduced.

## Conclusions

To our knowledge, this is the first study to show that dual-task test outcomes discriminate between groups of individuals with early-stage dementia diagnoses, MCI, SCI, and healthy controls. The novel TUGdt test outcomes “animals/10 s” and “months/10 s” demonstrate a high level of discrimination between the investigated groups. The use of these dual-task test outcomes implies consideration of both mobility and verbal performances, which should eliminate the possible effect of task prioritization. Thus, we conclude that both TUGdt NA and TUGdt MB have the potential to be used as a tool to assist in discriminating between individuals with dementia disorder, MCI, SCI, and no cognitive impairment. Possible areas of future clinical use would be either as a screening tool to conduct before a specialized memory assessment, or as a complementary test in the specialized memory assessment. Ongoing UDDGait studies will be focused on the possible predictive value of these TUGdt test outcomes and on the implementation of TUGdt testing in clinical practice.

## Supplementary information

**Additional file 1.** Additional assessments.

**Additional file 2.** Standardized odds ratios of Timed Up-and-Go and Timed Up-and-Go dual-task outcomes.

## Data Availability

The material analyzed during the current study is not publicly available due to its content of sensitive personal data. Datasets generated may be available from the principal investigator (ACÅ) on reasonable request, after ethical considerations.

## Abbreviations

AD: Alzheimer’s disease
MCI: Mild cognitive impairment
MMSE: Mini Mental State Examination
OR: Odds Ratio
SCI: Subjective cognitive impairment
TUG: Timed Up-and-Go
TUGst: Timed Up-and-Go single-task
TUGdt: Timed Up-and-Go dual-task
TUGdt NA: Timed Up-and-Go dual-task naming animals
TUGdt MB: Timed Up-and-Go dual-task months backwards
UDDGait: Uppsala Dalarna Dementia and Gait project
