# Supplementary material for: Dual-task tests discriminate between dementia, mild cognitive impairment, subjective cognitive impairment, and healthy controls – a cross-sectional cohort study
Source: BMC Geriatr. 2020 Jul 29;20:258. doi: 10.1186/s12877-020-01645-1 (PMC7392684; doi:10.1186/s12877-020-01645-1)
Supplement: Supplementary file 1 — Additional file 1. Additional assessments. [file 12877_2020_1645_MOESM1_ESM.docx]

| **Additional file 1. Additional Assessments** | | | | | |
| --- | --- | --- | --- | --- | --- |
| **Assessment** | **Total Sample**  **(N = 464)** | **Dementia Disorders**  **(n = 86)** | **MCI**  **(n = 135)** | **SCI**  **(n = 77)** | **Healthy Controls**  **(n = 166)** |
| GDS-4^a^, score, *Md* (IQR)  (Min.–max.) | 0 (0-1)  (0-4) | 0 (0-1)  (0-4) | 0 (0-1)  (0-4) | 0 (0-2)  (0-4) | 0 (0-0)  (0-4) |
| Bohannon balance^b^, score, *Md* (IQR)  (Min.–max.) | 6 (5-6)  (2-6) | 5 (5-6)  (2-6) | 5 (5-6)  (4-6) | 6 (5-6)  (4-6) | 6 (5-6)  (4-6) |
| GMF assistance, score, *Md* (IQR)  (Min.-max.) | 0 (0–0)  (0–7) | 0 (0–0)  (0–4) | 0 (0–0)  (0–2) | 0 (0–0)  (0–7) | 0 (0–0)  (0–2) |
| GMF pain, score, *Md* (IQR)  (Min.-max.) | 0 (0–0)  (0–7) | 0 (0–1)  (0–7) | 0 (0–0)  (0–7) | 0 (0–1)  (0–7) | 0 (0–0)  (0–5) |
| GMF insecurity, score, *Md* (IQR)  (Min.-max.) | 0 (0–0)  (0–7) | 0 (0–1)  (0–7) | 0 (0–0)  (0–6) | 0 (0–0)  (0–7) | 0 (0–0)  (0–4) |
| Hand grip^c^, pounds, *Md* (IQR)  (Min.-max.) | 70 (55-96)  (24-165) | 60 (48-83)  (25-128) | 74 (54-95)  (25-130) | 75 (60-110)  (34-146) | 73 (60-99)  (24-165) |

^a^Missing values, n = 2

^b^Missing values, n = 2

^c^Missing values, n = 26

MCI = mild cognitive impairment; SCI = subjective cognitive impairment; *Md* = Median; IQR = Interquartile range; Min. = minimum; Max. = maximum; GDS-4 = Geriatric Depression Scale (short version); GMF = General Motor Function Assessment Scale.
