# Supplementary material for: Dual-task tests discriminate between dementia, mild cognitive impairment, subjective cognitive impairment, and healthy controls – a cross-sectional cohort study
Source: BMC Geriatr. 2020 Jul 29;20:258. doi: 10.1186/s12877-020-01645-1 (PMC7392684; doi:10.1186/s12877-020-01645-1)
Supplement: Supplementary file 2 — Additional file 2. Standardized odds ratios of Timed Up-and-Go and Timed Up-and-Go dual-task outcomes. [file 12877_2020_1645_MOESM2_ESM.docx]

| **Additional file 2:** **Standardized Odds Ratios of Timed Up-and-Go and Timed Up-and-Go Dual-Task Outcomes** | | | | | |
| --- | --- | --- | --- | --- | --- |
| **Timed Up-and-Go Test Result** | **Outcome** | **Odds Ratio Estimate** | **Lower 95% CL for Odds Ratio** | **Upper 95% CL for Odds Ratio** | ***p*** |
| ***TUG single task, s** | Dementia Disorders vs. SCI | 2.50 | 1.56 | 4.01 | **0.001** |
|  | Dementia vs. Healthy Controls | 5.99 | 3.55 | 10.10 | **<.001** |
|  | MCI vs. Healthy Controls | 4.62 | 2.97 | 7.19 | **<.001** |
| ***TUGdt NA, s** | Dementia Disorders vs. SCI | 2.38 | 1.48 | 3.84 | **<.001** |
|  | Dementia Disorders vs. Healthy Controls | 3.76 | 2.42 | 5.84 | **<.001** |
|  | MCI vs. Healthy Controls | 2.68 | 1.83 | 3.93 | **<.001** |
| ***TUGdt MB, s** | Dementia Disorders vs. SCI | 2.85 | 1.73 | 4.67 | **<.001** |
|  | Dementia Disorders vs. Healthy Controls | 5.33 | 3.18 | 8.93 | **<.001** |
|  | MCI vs. Healthy Controls | 3.67 | 2.38 | 5.64 | **<.001** |
| ***TUGdt NA cost, %** | Dementia Disorders vs. SCI | 1.09 | 0.71 | 1.69 | 0.691 |
|  | Dementia Disorders vs. Healthy Controls | 0.86 | 0.61 | 1.22 | 0.392 |
|  | MCI vs. Healthy Controls | 0.72 | 0.54 | 0.95 | 0.021 |
| ***TUGdt MB cost, %** | Dementia Disorders vs. SCI | 1.34 | 0.94 | 1.91 | 0.109 |
|  | Dementia Disorders vs. Healthy Controls | 1.18 | 0.87 | 1.59 | 0.299 |
|  | MCI vs. Healthy Controls | 1.01 | 0.78 | 1.31 | 0.940 |
| **†TUGdt, number of animals** | Dementia Disorders vs. SCI | 3.55 | 2.16 | 5.84 | **<.001** |
|  | Dementia Disorders vs. Healthy Controls | 7.57 | 4.41 | 13.00 | **<.001** |
|  | MCI vs. Healthy Controls | 3.49 | 2.40 | 5.07 | **<.001** |
| **†TUGdt, number of months** | Dementia Disorders vs. SCI | 4.53 | 2.61 | 7.87 | **<.001** |
|  | Dementia Disorders vs. Healthy Controls | 10.77 | 5.84 | 19.85 | **<.001** |
|  | MCI vs. Healthy Controls | 3.98 | 2.72 | 5.82 | **<.001** |
| **†TUGdt, animals/10s** | Dementia Disorders vs. SCI | 6.03 | 3.12 | 11.65 | **<.001** |
|  | Dementia Disorders vs. Healthy Controls | 22.26 | 9.88 | 50.17 | **<.001** |
|  | MCI vs. Healthy Controls | 7.07 | 4.29 | 11.64 | **<.001** |
| **†TUGdt, months/10s** | Dementia Disorders vs. SCI | 6.52 | 3.40 | 12.50 | **<.001** |
|  | Dementia Disorders vs. Healthy Controls | 23.70 | 10.38 | 54.09 | **<.001** |
|  | MCI vs. Healthy Controls | 7.11 | 4.46 | 11.35 | **<.001** |

All results are adjusted for participant age, gender and educational level.

*Standardized odds ratios measure risk increase per one standard deviation *increase* of the predictor

**†**Standardized odds ratios measure risk increase per one standard deviation *decrease* of the predictor
